# Identification of Genetic Predispositions Related to Ionizing Radiation in Primary Human Skin Fibroblasts From Survivors of Childhood and Second Primary Cancer as Well as Cancer-Free Controls: Protocol for the Nested Case-Control Study KiKme

**DOI:** 10.2196/32395

**Published:** 2021-11-11

**Authors:** Manuela Marron, Lara Kim Brackmann, Heike Schwarz, Willempje Hummel-Bartenschlager, Sebastian Zahnreich, Danuta Galetzka, Iris Schmitt, Christian Grad, Philipp Drees, Johannes Hopf, Johanna Mirsch, Peter Scholz-Kreisel, Peter Kaatsch, Alicia Poplawski, Moritz Hess, Harald Binder, Thomas Hankeln, Maria Blettner, Heinz Schmidberger

**Affiliations:** 1 Leibniz Institute for Prevention Research and Epidemiology – BIPS Bremen Germany; 2 Department of Radiation Oncology and Radiation Therapy University Medical Center of the Johannes Gutenberg University Mainz Germany; 3 Department of Orthopedics and Traumatology University Medical Center of the Johannes Gutenberg University Mainz Germany; 4 Radiation Biology and DNA Repair Technical University of Darmstadt Darmstadt Germany; 5 Institute of Medical Biostatistics, Epidemiology and Informatics University Medical Center of the Johannes Gutenberg University Mainz Germany; 6 German Childhood Cancer Registry Institute for Medical Biostatistics, Epidemiology and Informatics University Medical Center of the Johannes Gutenberg University Mainz Germany; 7 Institute of Medical Biometry and Statistics Faculty of Medicine and Medical Center Freiburg University of Freiburg Freiburg Germany; 8 Institute of Organismic and Molecular Evolution, Molecular Genetics and Genome Analysis Johannes Gutenberg University Mainz Germany

**Keywords:** fibroblast, irradiation, childhood cancer, neoplasm, second primary neoplasm, second cancer, study design, participation, feasibility, cell line

## Abstract

**Background:**

Therapy for a first primary neoplasm (FPN) in childhood with high doses of ionizing radiation is an established risk factor for second primary neoplasms (SPN). An association between exposure to low doses and childhood cancer is also suggested; however, results are inconsistent. As only subgroups of children with FPNs develop SPNs, an interaction between radiation, genetic, and other risk factors is presumed to influence cancer development.

**Objective:**

Therefore, the population-based, nested case-control study KiKme aims to identify differences in genetic predisposition and radiation response between childhood cancer survivors with and without SPNs as well as cancer-free controls.

**Methods:**

We conducted a population-based, nested case-control study KiKme. Besides questionnaire information, skin biopsies and saliva samples are available. By measuring individual reactions to different exposures to radiation (eg, 0.05 and 2 Gray) in normal somatic cells of the same person, our design enables us to create several exposure scenarios for the same person simultaneously and measure several different molecular markers (eg, DNA, messenger RNA, long noncoding RNA, copy number variation).

**Results:**

Since 2013, 101 of 247 invited SPN patients, 340 of 1729 invited FPN patients, and 150 of 246 invited cancer-free controls were recruited and matched by age and sex. Childhood cancer patients were additionally matched by tumor morphology, year of diagnosis, and age at diagnosis. Participants reported on lifestyle, socioeconomical, and anthropometric factors, as well as on medical radiation history, health, and family history of diseases (n=556). Primary human fibroblasts from skin biopsies of the participants were cultivated (n=499) and cryopreserved (n=3886). DNA was extracted from fibroblasts (n=488) and saliva (n=510).

**Conclusions:**

This molecular-epidemiological study is the first to combine observational epidemiological research with standardized experimental components in primary human skin fibroblasts to identify genetic predispositions related to ionizing radiation in childhood and SPNs. In the future, fibroblasts of the participants will be used for standardized irradiation experiments, which will inform analysis of the case-control study and vice versa. Differences between participants will be identified using several molecular markers. With its innovative combination of experimental and observational components, this new study will provide valuable data to forward research on radiation-related risk factors in childhood cancer and SPNs.

**International Registered Report Identifier (IRRID):**

DERR1-10.2196/32395

## Introduction

Childhood cancer is defined as a malignant neoplasm or any neoplasm in the central nervous system occurring in children and adolescents before the age of 20 years [1]. Worldwide, the age-standardized incidence rate (world standard) is 152.8 per million person-years in those aged 0 to 19 years, is slightly higher in boys than in girls, and varies for different diagnostic groups dependent on age and region [2]. Risk factors for most childhood cancers remain largely unknown [1]. Common genetic susceptibility with low risk and rare genetic disorders with high risk explain less than 10% of the cases [3-15]. Corresponding with the current state of science, the immune system also plays an important role in the development of cancer [16], and several environmental factors [17-26], such as early infections [27] and vaccination [28], have been suggested but not established to be protective by modulating immunological pathways, in particular for childhood leukemia. In contrast, specific chemical substances such as benzene are established risk factors for the development of leukemia and antineoplastic agents (eg, DNA alkylating agents, topoisomerase II inhibitors, doxorubicin) for the development of acute myeloid leukemia and sarcomas in childhood [29]. However, these substances do not constitute the major part in the development of childhood cancer, since only a minority of children is exposed to such chemical carcinogens [30].

Exposure to high doses of ionizing radiation, either due to nuclear disasters [31] or in cancer therapies [32-35], is a rare and known environmental risk factor for acute myeloid leukemia in childhood [1] and second primary neoplasms (SPNs) [1,29,36-39,41]. Indeed gene-radiation interactions are assumed to be involved in the etiology of childhood cancer [1,42] and SPNs [43-46] as well. Besides high-dose ionizing radiation, the magnitude of the risk for first primary neoplasms (FPNs) in childhood from very low doses (≤0.05 Gray [Gy]) is still uncertain and difficult to resolve via conventional epidemiological studies [1]. Low doses of ionizing radiation are commonly used in medical diagnostics, like computed tomography examinations [47], and regarded as a risk factor in addition to the directly exposed treatment volume, where high doses of ionizing radiation are applied during radiation therapy [48]. Exposure to low doses also occurs during the staging procedure of neoplasms via computed tomography examinations and follow-up after treatment. In utero exposure to ionizing radiation during abdominal X-rays of pregnant women was consistently observed to be a risk factor for acute leukemia in many epidemiological studies conducted in the 1950s and 1960s [49-56]. Today, X-ray examinations during pregnancy are conducted using lower radiation doses [57], and recent studies were not able to identify any increased risk anymore [58]. Similarly, a recent study on cancer incidence after exposure to postnatal diagnostic X-rays did not find an increased risk for leukemia, lymphoma, central nervous system tumors, blastomas, or sarcomas [59]. However, data on the effect of low doses are still scarce and inconsistent due to missing direct biological human evidence [60,61]. Additionally, observational studies are often small and may not show proper confounder control [62-69].

To address these open questions and challenges with a more powerful approach, we designed a nested, molecular-epidemiological, case-control study that combines observational epidemiological research with standardized experimental components in primary human fibroblasts. We want to identify genetic predispositions related to the cellular response to high and low doses of ionizing radiation in SPN cases compared with FPN controls first and in childhood cancer cases compared with cancer-free controls second. This publication focuses on the description of the innovative study design and its potential use in research as well as on procedures of sampling and proportions of participation.

## Methods

### Aim and Study Design

The population-based, nested case-control study KiKme (German: “Krebserkrankungen im Kindesalter und molekulare Epidemiologie”; English: “Cancer in childhood and molecular-epidemiology”) was designed to analyze genetic predispositions and other molecular-biological factors associated with ionizing radiation in primary human fibroblasts from former childhood cancer patients (SPNs and FPNs) and cancer-free controls. Applying a molecular-epidemiological, case-control study design, using primary human skin fibroblasts as a model of normal human somatic tissue enables us to measure individual changes in reaction to different radiation exposures on a cellular level and to conduct an informed search for genomic causes in fibroblasts from the same person simultaneously [70]. The combination with observational data from questionnaires and the linkage of therapy data on chemo- and radiotherapy from treating hospitals complete the study and allow us to control for known confounding factors.

### Study Population

More than 70,000 former childhood cancer patients are registered in the German Childhood Cancer Registry [71]. This large cohort provides the basis for the nested case-control study KiKme. Since 1980, this registry has recorded population-based childhood cancer cases occurring in children younger than 15 years old in former Western Germany with almost complete coverage. Since 1991, cases from former Eastern Germany are recorded as well. In 2009, the age limit for recorded childhood cancer was raised from under 15 years old to under 18 years old [32]. Diagnoses of childhood cancer are validated in cooperation with treating hospitals and an open-end follow-up is conducted with an emphasis on obtaining information on SPNs [72]. The cohort in which our case-control study KiKme was nested includes children with only 1 cancer diagnosis (FPN) as well as with multiple cancer diagnoses over time (SPN). Subjects were eligible if they were diagnosed with an FPN in childhood, were at least 18 years old (as of June 2012), showed survival after cancer diagnosis for 1 year or more, and were still alive when the study was performed. Additionally, an address and an agreement for data storage in the German Childhood Cancer Registry had to be available. The inclusion criteria resulted in a maximum of 1976 available former childhood cancer patients (247 SPNs with 1729 matching FPNs). All these former childhood cancer patients were initially contacted by the German Childhood Cancer Registry in consideration with the guidelines of the Association for Pediatric Oncology and Hematology in Germany.

For the pilot study of this project, 48 former childhood cancer patients with any morphology of FPN and SPN were included. Within the main study period, only participants (n=392) with an FPN of the most common childhood cancers of the International Classification of Childhood Cancer - third edition (ICCC-3) [73] were recruited: leukemia ICCC-3 I(a), I(b), I(c), I(d); lymphoma ICCC-3 II(a), II(b), II(c); and tumors of the central nervous system ICCC-3 III(a), III(b), III(c), III(d), IV(a). Cancer sites of the second primary diagnosis had to be at a potentially radiation-related site: thyroid carcinoma ICCC-3 XI(b); skin carcinoma ICCC-3 XI(e); leukemia ICCC-3 I(a), I(b), I(d) (all causally related to radiation [41]); or malignant melanoma ICCC-3 XI(d) (potentially related to radiation [41]). The number of possible SPN cases meeting the inclusion criteria was limited by the quantity of potential SPN participants who were still alive (n=247). Potential FPN controls (n=1729) were matched by age at recruitment (maximal age range of 5 years), sex, cancer morphology (ICCC-3), year of diagnosis (maximal range of 7 calendar years), and age at diagnosis (maximal age range of 4 years) to available SPN cases using a risk set sampling approach. Taking the year of diagnosis into account enables us to control for changes in therapy procedures. To be included as a possible FPN control, no SPN diagnosis had to exist at the date of the second diagnosis of the corresponding SPN case, and the FPN control had to be alive.

In order to not only be able to compare genetic predispositions related to ionizing radiation in SPN cases and FPN controls, we also recruited cancer-free controls for each matching group in an additional hospital-based study arm in the Department of Orthopedics and Traumatology of the University Medical Center Mainz. They were matched by sex and within a maximal 10-year age range at the time of the recruitment to participating SPN cases and FPN controls. Cancer-free controls were mainly recruited from patients who were hospitalized for elective orthopedic surgery after an accident. Cancer-free controls with severe or chronic diseases (eg, cancer, Alzheimer’s disease, multiple sclerosis, cardiovascular disease, diabetes) were excluded from participation due to a possible association with shared genetic predispositions and cancer development [74].

### Procedures and Survey Modules

The study combines information from questionnaires and molecular-biological experiments including investigations on radiation-induced effects using primary human skin fibroblasts derived from skin biopsies of the participants. In addition, saliva samples were collected as a second, independent source for DNA. Participants who reported being infected with severe infectious diseases (eg, hepatitis or AIDS) were excluded from a skin biopsy and saliva collection to avoid any transmission in the laboratory. Also, skin biopsies were not conducted if participants suffered from other severe diseases (eg, hemophilia) to prevent them from suffering adverse health consequences.

#### Questionnaires

Most study participants (SPN, FPN, cancer-free control) answered a self-completed questionnaire to assess socioeconomic and anthropometric factors, as well as information on lifestyle, medical history, and health. The general questionnaire contained questions on birth characteristics, ethnic origin, anthropometric factors, education, current life circumstances, smoking, drinking, diseases, and medications, as well as medical therapies and lifelong exposure to medically applied radiation (medical radiation history) of the participant. Data on cancer therapies were validated by comparing questionnaire data with information on type and dose of medication as well as dose and number of radiotherapy fractions from therapy protocols of treating hospitals [75]. All therapy data will be used to develop an individual exposure matrix for each participant. Furthermore, there were questions on family history of severe diseases. The complex information on family history of cancer was additionally requested in a personal interview in the clinic or through a telephone interview for all participants not attending the clinic in Mainz. The interview included information about cancer type and age at diagnosis within their relatives (children, siblings, nephews and nieces, parents, grandparents, aunts, uncles, and cousins).

#### Saliva Collection, Processing, and Storage

Saliva collection took place using the Oragene DNA Kit (DNA Genotek Inc, Ottawa, Ontario, Canada). The participant was asked not to drink, eat, smoke, or chew chewing gum 30 minutes before collection. Five minutes before the start, the participant rinsed his or her mouth and filled the saliva tube of the kit with saliva without air bubbles. The saliva was mixed with the DNA stabilizing fluid and immediately forwarded to the laboratory within the recruitment center. For persons participating near their residence, saliva samples were sent to the laboratory in Mainz in a provided cardboard box by standard mail. After receiving the collected samples, half of each saliva sample was lysed and incubated at 56 °C in the laboratory. After incubation, samples were mixed with ethanol, and the lysate was loaded in a NucleoSpin Blood L Column and centrifuged. After washing the silica membrane, the DNA was eluted with DNA buffer. The DNA sample was then stored at –80 °C. The remaining half of saliva from each participant was stored at –20 °C for later use.

#### Skin Biopsy Collection, Processing, and Storage

Skin samples were taken by punch biopsy under local anesthesia with a diameter of 3 mm at the cubital region for cancer patients and during surgery in the scar region for cancer-free controls. The resulting wounds were sewn with a single stitch. After successful extraction, biopsied skin was transferred to a vial with rich cell culture medium (Amniogrow, CytoGen GmbH, Wetzlar, Germany), stored at room temperature, and immediately taken to the laboratory or by courier service within 24 hours. Subcutaneous tissue was removed, and the biopsy was dissected in rich cell culture medium (Amniogrow, CytoGen GmbH, Wetzlar, Germany) and cultured in a humidified incubator at 37 °C with 5% CO_2_ (Heracell Vios 160i, Thermo Fisher Scientific, Waltham, MA) to allow the outgrowth and expansion of fibroblasts. Culture medium (Amniogrow, CytoGen GmbH, Wetzlar, Germany) was changed every 3-4 days. Passaging of fibroblasts was done using 0.05% trypsin with 0.1% ethylenediaminetetraacetate when reaching approximately 70% confluence. After the first passage, cells were cultured in low glucose Dulbecco’s minimal essential medium (Sigma-Aldrich, St. Louis, MO) containing 1% nonessential amino acids, 15% fetal bovine serum, and 1% penicillin/streptomycin (all supplements from Biochrom GmbH, Berlin Germany). Cultures were grown for 2-4 weeks to reach sufficient cell numbers for cryopreservation in liquid nitrogen or nitrogen gas.

### Sampling

All applicable institutional and governmental regulations concerning the ethical use of human volunteers were followed during this research. Approval by the Ethics Committee of the Medical Association of Rhineland-Palatinate was obtained (no. 837.262.12 (8363-F), no. 837.103.04 (4261), and no. 837.440.03 (4102)). Study participants who voluntarily gave consent for examinations, collection of samples, subsequent analysis, time-limited storage of personal data, and collected samples were included. Participants could consent to single components of the study while abstaining from others at any time. After confirmation to participate in the KiKme study, an appointment for the discussion of the informed consent was made. A date for skin biopsy, saliva sampling, and telephone or personal interview was obtained. Cases participating at the University Medical Center Mainz were offered the possibility of medical consultation. These consultations were not documented for this report. Participants were reimbursed and compensated for travel costs. To further increase participation despite potential long travel to Mainz, all cancer patients were also given the option to participate near their residence. If available, participants could name their attending dermatologist. Otherwise, the study team contacted a dermatologist near the residence of the participant. The attending dermatologists were asked to act as a cooperating partner, were trained for the study, and took the skin biopsy with the signed informed consent.

Potential cancer-free control participants were identified in the surgery schedules of the department for orthopedic surgery. They were contacted and informed about the content of the study during their stay in the hospital. Participation could be refused at any time during the procedure. To increase the study participation of cancer-free controls, the biopsy was taken from excess material during their surgical procedure.

### Analysis Plan

From all participants, cultured human fibroblasts from 156 participants with the best matching results based on our criteria (52 triplets each with 1 SPN, 1 FPN, and 1 cancer-free control participant) will be selected for the radiation experiments (mean age of participants at sampling: SPN 33 years, range 20-51 years; FPN 33 years, range 21-49 years; controls 33 years, range 19-48 years; median age of participants at first neoplasm: SPN 8 years, range 0-14 years and FPN 8 years, range 1-14 years; mean calendar year of the first neoplasm: SPN 1991, range 1980-2011 and FPN 1991, range 1980-2009). During radiation experiments, cultured human fibroblasts from each of the 156 selected and carefully matched participants will be exposed to a low (eg, 0.05 Gy) as well as a high dose (2 Gy) of X-rays and will be sham-irradiated (0 Gy). The low dose of radiation will be applied to mimic an exposure scenario during medical diagnostics (eg, computed tomography), and the high dose represents an average single tumor dose applied to the target volume of conventional fractionated radiation therapy. The fibroblast of each triplet will be treated simultaneously to avoid batch effects within groups. In a preliminary analysis, we identified the time point after radiation with the highest amount of differentially expressed genes for our chosen radiation doses [76]. The identified time point will be used to analyze differences in gene expression patterns between patient groups. The high number of samples from different participants in irradiation experiments (around one-third of the participants) allows us to distinguish possible gene expression patterns with candidate genes and underlying cellular pathways between groups and to identify differences between SPN cases and FPN controls as well as differences between former childhood cancer patients (SPNs and FPNs) and cancer-free controls. To be able to compare gene expression before and after exposure to ionizing radiation, RNA from 468 dishes with cultured human fibroblasts of the irradiation experiments (156 exposed to 0.05 Gy, 156 exposed to 2 Gy, and 156 sham-irradiated; 3 dishes for each participant) will be extracted and Illumina-sequenced. RNA sequencing data will be processed and cleaned as well as normalized using the Voom method [77]. Gene expression of irradiated cells will be compared with the expression of sham-irradiated cells after the same time interval for each participant. Differentially expressed genes dependent on radiation dose will be detected using linear models and empirical Bayesian statistics. The differential gene expression after irradiation will be computed by comparing measurements of fibroblasts from each participant with measurements after sham-irradiation (eg, counts of transcripts in cells of each individual after 0 Gy versus counts after 2 Gy). *P* values will be computed for the interaction between the effect of radiation and group and for the effect of radiation alone using the R package limma (lmFit, eBayes, makeContrasts) with *patient ID* as a block variable and the factors *patient group* and *radiation doses* [78]. The analyses will be performed without adjustment, with adjustment for age only, and with adjustment for age and gender. For the comparison between former childhood cancer patients with and without SPNs, the analyses will additionally be adjusted for age at first primary neoplasm diagnosis and for tumor subtype. Furthermore, sensitivity analyses will be performed separately for male participants and female participants with age adjustment. Differentially expressed genes will then be selected at a false discovery rate (FDR) level of 0.05 (Benjamini-Hochberg procedure). In addition, differentially expressed genes and their log_2_ fold change will be examined using Ingenuity Pathway Analysis (IPA; Version 1.13, QIAGEN Inc, 2018) with a right-tailed Fisher exact test examining pathway enrichment and z-score (≥|2|) indicating (in-) activation of pathways [79]. In addition, IPA will be employed to predict upstream regulators as well as downstream diseases and functions. We will choose promising marker genes to validate the RNA sequencing experiments via real-time quantitative polymerase chain reaction. Thus, RNA sequencing data intend to identify differentially expressed candidate genes, which finally enables a weighted analysis of DNA single-nucleotide variants (SNVs) in these genes and related regions by selecting the smallest *P* value from all comparisons. To filter SNVs, a gene list will be created that contains all genes that were identified as differentially expressed in the messenger RNA and long noncoding RNA analyses after Bonferroni correction (with adjustment for age and gender as well as with adjustment for age at first tumor diagnosis and for tumor type). Furthermore, the list could be supplemented with genes from the associated pathways of the Ingenuity Pathway Database and known radiation-associated genes (RadAtlas) [80] as well as genes associated with childhood cancer (International Cancer Genome Consortium [ICGC], Pediatric Cancer Genomic Data Portal [PeCan], PedcBio portal, Pediatric cancer gene database [Pedican], Xena browser) [81,82]. SNVs will be assigned to the genes if they are located in an area that includes the gene body, consisting of exons and introns, and 500 kilobases upstream and downstream of the gene body. In addition, SNVs will be assigned to the genes that were identified in the Genotype-Tissue Expression (GTEx) project [83] as expression quantitative trait loci (eQTLs) for the gene [84]. The analysis will be carried out using forest tests (RVTEST) [85,86] applying a single-variant Wald test at the SNV level. The burden test (combined multivariate and collapsing [CMC] method) [87], sequence kernel association test (SKAT) [88], and variable threshold method [89] will be used for the gene-based examination of the DNA sequencing data at RVTEST. Association studies will be performed based on the generated gene list using FDR as correction for multiple testing with a significance level of 5% and genome wide without FDR adjustment. Simulation studies assuming our sample size and different SNV effect sizes (odds ratio [OR] 1.3, 1.5, 2, 3, and 4) for genome-wide association studies resulted in the significance level selection of 5% at the gene level and 0.005% at the SNV level. In addition, a weighted analysis of SNVs will be performed genome wide by using likelihood-based boosting [85] and gene list *P* values as weights. Both tumor groups (former SPN and FPN patients) will be compared against the cancer-free controls, and, additionally, the tumor groups will be compared against each other. Results of the SNV analysis will be verified in a 2-stage procedure: First, identified genetic group differences in fibroblasts from about one-half of the participants (n=286) will be replicated in DNA sequenced from the saliva of the same participants. In the second stage, validated results will be replicated in the saliva DNA of an independent confirmation collective consisting of the remaining half of the participants (n=275). This 2-stage approach enables us to ameliorate problems of false discovery. Possible confounding or effect modification (eg, by sex, age at diagnosis of first or second primary neoplasm, type of first or second primary neoplasm, or batch effects) will be taken into account in this analysis. In addition, sensitivity analysis for other possible confounding factors like family history of cancer or received therapies will be conducted.

To identify possible risk associations with cancer treatment, participants were asked whether they had received cancer therapies. Used medications and affected body regions will be additionally inquired (n=556). For validation, self-reports will be compared with data from cancer therapies of the patients from hospitals and clinical studies [75]. By measuring sensitivity and specificity, the quality of binary variables will be analyzed. Receiver operatic characteristic curves will be used for a graphical comparison. Positive and negative predictive values will be used to analyze the validity of the questionnaire. Cohen kappa will be used to measure the concordance between the information from questionnaires and from treating hospitals. Influencing factors (eg, number of neoplasms, sex, sociodemographic factors, comorbidities, time since cancer treatment) on the dichotomous outcome variable *degree of agreement* will be analyzed using logistic regression [75]. If the questionnaire is reliable, conditional logistic regression and mixed models will be used to estimate possible risk associations with cancer therapies.

Differences in family history between childhood cancer patients with FPNs and SPNs as well as cancer-free controls could also be a confounder or effect modifier and will be investigated concerning family history of cancer, degree of family relatedness, age of diagnosis, and family history of chronic disease (n=556). Our interest here is to identify whether an increased number of cancer cases in families is associated with childhood cancer incidence. A family history of cancer was recorded as dichotomous variables for each degree of kinship, for maternal and paternal kinship, and for sex of family members in the questionnaires. The number of cases within families will be related to family size. Clustering of cancer within families will be estimated by the genealogical index of familiarity [90] and stratified by groups (SPN, FPN, cancer-free controls) to ascertain whether the average kinship among affected individuals in a pedigree differed from a randomly drawn control set of that pedigree. The kinship sum test [91] will be applied to identify affected individuals exhibiting a closer relationship to other affected individuals than would be expected by chance. Conditional logistic regression will be applied to investigate the association between family history of cancer and the risk of primary childhood cancer (SPN and FPN). Analyses will be adjusted for sex and age at recruitment and stratified for kinship and sex. Cox proportional hazard models will be calculated adjusted for age, sex, family history of cancer, and primary childhood tumor entity to estimate standard incidence rates for SPNs among the cohort of childhood cancer patients. Further, conditional logistic regressions will be used to explore the associations between childhood cancer (SPN and FPN) and other diseases in the family (eg, diabetes, hypertension, elevated blood cholesterol).

The available biosamples of the study will further be used to forward research on other biological markers (eg, hyper- and hypovariability of gene expression, noncoding RNA, copy number variations, epigenetic changes like methylation pattern of genes, proteins associated with double-strand breaks, chromosomal aberrations) and to investigate their possible association with radiation-related cancer development in other KiKme research projects.

## Results

**Figure 1:**
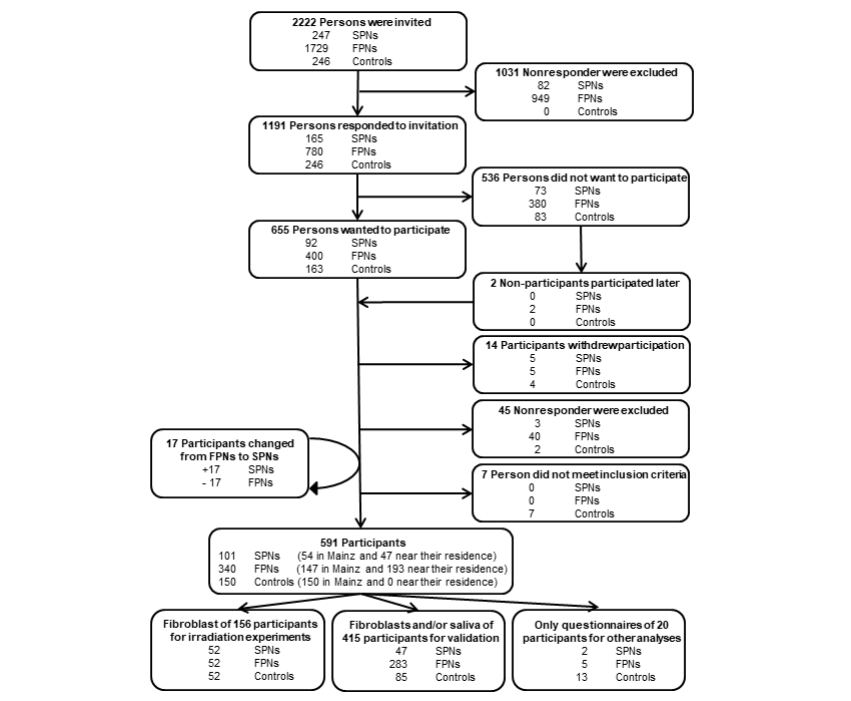
Enrollment of participants (SPNs, FPNs, and controls) in the population-based, nested case-control study KiKme. FPN: first primary neoplasm; SPN: second primary neoplasm.

The recruitment started in 2013, and the result is shown in Figure 1. Originally, we invited 247 SPNs and 1729 FPNs to participate in the study, of which 92 SPNs (92/247, 37.3%) and 399 FPNs (399/1729, 23.1%) were willing to participate. During the recruiting process, some participants refused their participation while others accepted. Thus, some rematching was needed. To gain complete matching groups in the radiation experiments, we allowed 17 FPN patients that developed an SPN later in life to migrate to the SPN group. However, taking the risk set sampling approach into account, their questionnaire data could be used both as an SPN case and as an FPN control in the questionnaire-based analyses (eg, on the risk of family history of cancer). Overall, 54.4% of the participants (47 SPN and 193 FPN of 441 total participants) participated in the study near their residence in a medical practice of 1 of the 182 cooperating dermatologists.

A total of 591 former childhood cancer patients and cancer-free controls aged 19 to 53 years (mean age 32 years, 51% women and 49% men) participated in the study (Table 1). The age distribution of participants with SPNs compared with FPNs was very similar (χ² test: *P*=.28), whereas participating cancer-free controls were slightly younger than participants with childhood cancer (χ² test: *P*<.001). Similar differences were found for nonparticipating childhood cancer survivors and nonparticipating cancer-free controls (χ² test nonparticipants with SPNs versus FPNs: *P*=.11; χ² test nonparticipating cancer-free controls versus nonparticipating childhood cancer survivors: *P*<.001). Further characteristics of participants and nonparticipants like age at diagnosis and tumor morphology are summarized in Table 1 and Table 2.

**Table 1:** Characteristics of included study participants and nonparticipants.

Characteristics	Participants				Nonparticipants^a^			
	SPNs^b^ (n=101)	FPNs^c^ (n=340)	Controls (n=150)	Total (n=591)	SPNs (n=146)	FPNs (n=1389)	Controls (n=96)	Total (n=1631)
Female, n (%)	50 (49.5)	189 (55.6)	62 (41.3)	301 (50.9)	71 (48.6)	606 (43.6)	42 (43.8)	719 (44.1)
Male, n (%)	51 (50.5)	151 (44.4)	88 (58.7)	290 (49.1)	65 (44.5)	657 (47.3)	54 (56.2)	776 (47.6)
Sex missing, n (%)	N/A^d^	N/A	N/A	N/A	10 (6.8)	126 (9.1)	0 (0)	136 (8.3)
Age at recruitment (years), mean (range)	32 (19-51)	34 (19-53)	29 (18-48)	32 (19-53)	34 (18-49)	34 (18-51)	31 (18-51)	33 (18-51)
<25 years old, n (%)	19 (18.8)	44 (12.9)	57 (38.0)	120 (20.3)	18 (12.3)	111 (8.0)	17 (17.7)	146 (9.0)
25-29 years old, n (%)	25 (24.8)	69 (20.3)	40 (26.7)	134 (22.7)	18 (12.3)	234 (16.8)	25 (26.0)	277 (17.0)
30-34 years old, n (%)	19 (18.8)	78 (22.9)	20 (13.2)	117 (19.8)	24 (16.4)	245 (17.6)	19 (29.8)	288 (17.7)
≥35 years old, n (%)	38 (37.6)	149 (43.8)	33 (22.0)	220 (37.2)	75 (51.4)	672 (48.4)	30 (31.3)	777 (47.6)
Age missing, n (%)	N/A	N/A	N/A	N/A	11 (7.5)	127 (9.1)	5 (5.2)	143 (8.8)
Age at 1st diagnosis (years), mean (range)	7 (0-14)	8 (0-16)	N/A	N/A	8 (0-14)	7 (0-15)	N/A	N/A
Year of 1st diagnosis	1980-2011	1980-2012	N/A	N/A	1980-2005	1980-2012	N/A	N/A
Years between 1st and 2nd diagnoses, mean (range)	16 (2-35)	N/A	N/A	N/A	16 (1-30)	N/A	N/A	N/A
Age at 2nd diagnosis (years), mean (range)	23 (5-46)	N/A	N/A	N/A	24 (5-41)	N/A	N/A	N/A
Year of 2nd diagnosis	1986-2018	N/A	N/A	N/A	1989-2014	N/A	N/A	N/a

^a^Information available only for nonparticipants from the main study.

^b^SPNs: second primary neoplasms.

^c^FPNs: first primary neoplasms.

^d^N/A: not applicable.

**Table 2:** Cancer sites and cancer therapies of the included study participants and nonparticipants.

Cancer site (International Classification of Childhood Cancer 3rd Edition)		Participants			Nonparticipants^a^	
		SPNs^b^ (n=101)	FPNs^c^ (n=340)	SPNs (n=146)		FPNs (n=1389)
**1st neoplasm, n (%)**						
	Leukemia (I(a), I(b), I(c), I(d))	41 (40.6)	166 (48.8)	66 (45.2)		641 (46.1)
	Lymphoma (II(a), II(b), II(c))	41 (40.6)	135 (39.7)	40 (27.4)		485 (34.9)
	Central/peripheral nervous system (III(a), III(b), III(c), III(d), IV(a))	15 (14.9)	35 (10.3)	29 (19.9)		138 (9.9)
	Other tumors V, VI(a), IX(a), IX(e)	4 (4.0)	4 (1.2)	0 (0.0)		0 (0.0)
**2nd neoplasm, n (%)**						
	Thyroid cancer (XI(b))	30 (29.7)	N/A^d^	55 (37.7)		N/A
	Skin carcinoma (XI(e))	32 (31.7)	N/A	53 (36.3)		N/A
	Malignant melanoma (XI(d))	4 (4.0)	N/A	11 (7.5)		N/A
	Leukemia (I(a), I(b), I(d))	9 (8.9)	N/A	16 (11.0)		N/A
	Lymphoma (II(a), II(b))	6 (5.9)	N/A	N/A		N/A
	Central nervous system (III(a), III(b), III(e))	9 (8.9)	N/A	N/A		N/A
	Breast cancer (XI(f))	3 (3.0)	N/A	N/A		N/A
	Other unspecific carcinoma (XI(f))	6 (5.9)	N/A	N/A		N/A
	Sarcoma (IX(d), IX(e))	2 (2.0)	N/A	N/A		N/A
**3rd neoplasm, n (%)**						
	Renal carcinomas (VI(b))	1 (1.0)	N/A	—^e^		—
	Skin carcinoma (XI(e))	2 (2.0)	N/A	—		—
	Breast cancer (XI(f))	1 (1.0)	N/A	—		—
	Other and unspecified carcinomas (XI(f))	2 (2.0)	N/A	—		—
	Other specified intracranial and intraspinal neoplasms (III(e))	2 (2.0)	N/A	—		—
**4th neoplasm, n (%)**						
	Thyroid cancer (XI(b))	1 (1.0)	N/A	—		—
**Cancer therapies** **for the 1st neoplasm, n (%)**						
	Chemotherapy	93 (92.1)	312 (91.8)	—		—
	Radiation therapy	74 (73.3)	225 (66.2)	—		—
	Surgery	25 (24.8)	64 (18.8)	—		—
**Cancer therapies** **for the** **2nd neoplasm, n (%)**						
	Chemotherapy	22 (21.8)	N/A	—		—
	Radiation therapy	21 (20.8)	N/A	—		—
	Surgery	56 (55.4)	N/A	—		—
**Cancer therapies** **for the** **3rd neoplasm, n (%)**						
	Chemotherapy	1 (1.0)	N/A	—		—
	Surgery	2 (2.0)	N/A	—		—
**Cancer therapies** **for the** **4th neoplasm, n (%)**						
	Surgery	1 (1.0)	N/A	—		—

^a^Information available only for nonparticipants from the main study.

^b^SPNs: second primary neoplasms.

^c^FPNs: first primary neoplasms.

^d^N/A: not applicable.

^e^Information on 3rd and 4th diagnoses were obtained only from participants; therefore, this information is not available for nonparticipants.

For 95% (87/91) of participating SPN cases, suitable FPN controls with a maximum difference of 3 calendar years between first diagnoses could be identified (Multimedia Appendix 1). For the remaining 5%, the time difference was increased to 4-7 calendar years. The matching rate was comparable to the age at first diagnosis: 98% of SPN cases and FPN controls were diagnosed within 3 years of age, and 100% were diagnosed within 4 years of age. Matching for age at recruitment was accomplished within a 3-year age range for 93% (85/91) of participating SPN cases and FPN controls. The remaining 7% were matched by a maximum age range of 5 years. For 7 SPN cases (7/101, 6.9%), no suitable FPN cases participated in the study. However, their information from genetic analyses and questionnaires as well as the information from all other incomplete matching groups will also be included in the analyses.

Cancer-free controls (n=150) were recruited during their stay in the orthopedic surgery department and matched by age and sex to participating SPN cases and FPN controls. Participation proportion for cancer-free controls was originally 66.3% (163 participants of 246 directly contacted persons), but 6 cancer-free controls were excluded due to cancer diagnoses, 4 cancer-free controls actively withdrew from participation during the study period, 2 had to be excluded due to nonresponse, and 1 was excluded due to diabetes (Figure 1). An additional cancer-free control took part in both the pilot study and the main study, and therefore, this participant was excluded from the pilot data.

The difference in age at recruitment for participating SPN cases and cancer-free controls was not larger than 3 years for 95% (76/81) of cancer-free controls and not more than 5 years for 98% (79/81; Multimedia Appendix 1). Only 2 cancer-free controls (2/81, 2%) could not be matched within this age range. Included controls had a short hospital stay due to injuries or their consequences (87/150, 58.0%), joint diseases (17/150, 11.3%), osteopathy and chondropathy (14/150, 9.3%), diseases of the soft tissue (9/150, 6.0%), arthrosis (6/150, 4.0%), orthopedic after treatments (2/150, 1.3%), diseases of the skin and subcutaneous tissue (2/150, 1.3%), congenital malformations or deformities of the musculoskeletal system (1/150, 0.7%), diseases of the musculoskeletal system and connective tissue (1/150, 0.7%), or diseases of nerves, nerve roots, and nerve plexus (1/150, 0.7%). For 6.7% (10/150) of controls, no reason for the hospital stay was given.

Taking group changes from FPN to SPN into account, final participation proportions were 40.9% (101 participants out of 247 invited persons) for SPN cases, 19.7% (340 participants out of 1729 invited persons) for FPN controls, and 61.0% (150 participants out of 246 contacted persons) for cancer-free controls (Table 1). Mentioned reasons for refusal to participate were lack of interest or perceived lack of personal benefit (7 SPN, 49 FPN, 34 cancer-free controls), expenditure of time (36 SPN, 130 FPN, 14 cancer-free controls), illnesses (12 SPN, 20 FPN, 5 cancer-free controls), fear of skin biopsy (12 SPN, 50 FPN, 14 cancer-free controls), and unavailability due to insufficient language skills or problems of comprehension or incorrect contact information (1 SPN, 6 FPN, 5 cancer-free controls). All other participants (1235/1631, 75.7%) provided no reason for their refusal to participate.

In summary, this study successfully obtained questionnaire data for 85 SPN cases (84.2% of 101 participating SPN), 325 FPN controls (95.6% of 340 participating FPN), and 146 cancer-free controls (97.3% of 150 participating cancer-free controls). Skin biopsies were available from 92 SPN cases (91.1% of 101 participating SPN), 307 FPN controls (90.3% of 340 participating FPN), and 100 cancer-free controls (66.7% of 150 participating cancer-free controls). Overall, 3886 cryogenic tubes with primary skin fibroblasts were cryopreserved in liquid nitrogen for further experiments with a mean of 6.8 tubes per participant (SD 4.2, range: 0-28). In total, saliva samples were dispensed from 84 SPN cases (83.2% of 101 participating SPN) and 319 FPN controls (93.8% of 340 participating FPN), as well as from 108 cancer-free controls (72.0% of 150 participating cancer-free controls). Only 2 SPN cases, 3 FPN controls, and 13 cancer-free controls were unwilling to provide any biosamples for RNA and DNA analyses. Further, 2 FPN controls were excluded from the extraction of biosamples because of former hepatitis infections. Details on available survey modules and biosamples for participants are shown in Table 3 for each donor group.

**Table 3:** Actual available survey modules and biosamples for participants in each donor group.

Type of data		SPNs^a^ (n=101)	FPNs^b^ (n=340)	Controls (n=150)	Total (n=591)
**Questionnaire data, n (%)**					
	Participant information	85 (84.2)	325 (95.6)	144 (96.0)	554 (93.7)
	Family history of diseases	85 (84.2)	325 (95.6)	146 (97.3)	556 (94.1)
	Both questionnaires	85 (84.2)	325 (95.6)	144 (96.0)	554 (93.7)
**Biosamples, n (%)**					
	Biopsy	92 (91.1)	307 (90.3)	100 (66.7)	499 (84.4)
	Saliva	84 (83.2)	319 (93.8)	108 (72.0)	511 (86.5)
	Biopsy and saliva	77 (76.2)	291 (85.6)	71 (47.3)	439 (74.3)
	Biopsy or saliva	99 (98.0)	335 (98.5)	137 (91.3)	571 (96.6)
	No bio-samples	2 (2.0)	5 (1.5)	13 (8.7)	20 (3.4)
**Cryopreserved tubes of fibroblasts**					
	Total, n	757	2179	950	3886
	Tubes per participant, mean (SD)	7.7 (4.3)	6.5 (3.1)	6.9 (5.9)	6.8 (4.2)
	Tubes per participant, minimum	0	0	0	0
	Tubes per participant, maximum	20	16	28	28
**DNA extracts, n (%)**					
	From fibroblasts	90 (89.1)	301 (88.5)	97 (64.7)	488 (82.6)
	From saliva	84 (83.2)	319 (93.8)	107 (71.3)	510 (86.3)

^a^SPNs: second primary neoplasms.

^b^FPNs: first primary neoplasms.

## Discussion

### Principal Findings

Our molecular-epidemiological study is the first attempting to analyze observational data from questionnaires and molecular-biological factors associated with ionizing radiation in primary human fibroblasts of a unique childhood cancer survivor cohort. To study molecular-biological factors, we succeeded in obtaining fibroblasts derived from 499 skin biopsies and 511 saliva samples of former childhood cancer patients (SPNs and FPNs) and cancer-free controls. With this source, we can measure individual reactions to ionizing radiation in primary human skin fibroblasts. We will use these data for an informed analysis of potential genetic predispositions. Predispositions defined through DNA mutations can be identified using the DNA extracted from fibroblasts as well as saliva samples. Combining these results with observational data from questionnaires allows us to control for several confounding factors. During the recruitment process, we invited all former SPN and matched FPN patients from the German Childhood Cancer Registry who met our inclusion criteria. However, the number of eligible former childhood cancer patients was limited to 1990 even in such a large and long-running childhood cancer survivor cohort. While the participation of cancer-free controls was high (61%), the rate of participation among former childhood cancer patients was rather low (SPN 41%, FPN 20%). Different participation proportions can be explained by the nature of this study’s sampling strategy. Cancer-free controls were contacted in the hospital before undergoing surgery. Biopsies were then taken during that procedure without further effort for the patient. In contrast, SPN and FPN patients needed to travel or keep set appointments made for the biopsy. In general, the study involved complex logistics and high time expenditure for participants, especially for SPN and FPN participants. By implementing the possibility for former childhood cancer patients to participate near their residence, we reduced their effort and time spent on recruitment to a minimum. Our design required immense efforts in recruitment and data collection for the study centers. These efforts were worthwhile as they increased the rate of participation, even though an invasive procedure, such as skin biopsy, was demanded from more or less healthy individuals, and individual genetic analyses were performed. In summary, our study provides a new way of exploring the interplay between childhood cancer and second primary cancer predisposition and ionization radiation. We hope that this study will set a precedent and encourage others to perform similar projects on the international scale, requiring primary fibroblasts for experiments from large childhood cancer survivor cohorts and to investigate the underlying reasons for childhood cancer. This would help to improve therapeutic strategies, reduce the risk of developing a second primary cancer, and enhance the quality of the patients’ lives.

To identify molecular mechanisms potentially related to radiation and the development of childhood cancer, analyses at different levels are required to increase our knowledge. On the genomic level, single nucleotide polymorphisms (SNPs) can and should be analyzed in a population-based sample as it is common in genome-wide association studies (GWAS). Our sample size is limited by the number of available SPN cases and thus corresponds more to the size of a clinical cohort, which does not allow direct transfer of a GWAS approach. However, such clinical cohorts often consider gene expression and less frequently SNPs, which makes direct transmission difficult [92]. Additionally, the investigation of radiation-induced effects will be carried out experimentally by gene expression measurement before and after irradiation. To investigate the connection between radiation and childhood cancer, statistical techniques from these 3 perspectives — GWAS, clinical cohorts, and experiments — must be combined. With this combination, an increase in statistical power can be achieved. However, sufficient statistical power will still be limited to strong associations.

### Strengths and Limitations

In contrast to previously conducted studies that investigated the association between ionizing radiation and cancer risk [35,62-69,93-106], this epidemiological study is one of the first enabling the collection of detailed molecular-biological information before and after exposure of primary fibroblasts from a large number of participants exposed to diagnostic and therapeutic doses of ionizing radiation to investigate innate genetic radiation responses in the patients’ normal somatic cells [60,61]. We chose to perform experiments with primary fibroblasts, although lymphocytes used in other studies [107] would have been easier to attain by venipuncture. However, their survival and prolonged cultivation without immortalization by Epstein-Barr virus transformation are very limited [108]. Moreover, as some of our SPN and FPN donors have received bone marrow transplants, blood samples would have contained foreign blood cells of the bone marrow donors [109], which makes it impossible to analyze germline mutations of included cases. By measuring individual reactions to different exposures of radiation in normal somatic cells of the same person, our design enables us to create several exposure scenarios for the same participant simultaneously and therefore to trick the problem of counterfactual thinking and to avoid some confounding and bias [70]. The combination with observational data from questionnaires on medical radiation history, health, and family history of diseases allows comprehensive control for important confounders in the development of cancer. With additional collection of saliva samples from participants, DNA from an independent source is available for the validation and replication of results.

There are also several limitations to our study design. Given that we will analyze primary fibroblasts as monolayer cell cultures in vitro, this approach does not allow consideration of nontargeted radiation responses, such as the intercellular transmission of primarily adverse radiation effects to unirradiated neighboring cells via the so-called bystander effect, and their role in the development of therapy-related SPN [110]. Thus, the complexity of the 3D interaction of the in vivo radiation response and its clinical manifestation cannot be adequately represented by experiments in our study with monolayers of a single cell type. In addition, gene expression and radiation response of the chosen primary fibroblasts might not be representative of cells of various target organs and all cancer subtypes. However, the experiments conducted in this study enable first and very important insights into the etiology of childhood cancer and SPN. Moreover, the biological endpoints of this study might be influenced by the exposure history of the fibroblasts to possible carcinogenic factors (eg, cancer therapy, alcohol, tobacco, medication). To deal with this problem, our questionnaires cover a broad spectrum of possible confounding factors and allow us to control for them. As with all epidemiological studies requiring biological material from patients, our study underlies an inherent survivor bias, as solely living patients could be recruited. Severe cases with high mortality (eg, acute myeloid leukemia after acute lymphoid leukemia or 2 diagnoses in rapid succession) cannot be captured to a full extent by this study. A selection bias cannot be ruled out in this study, as individuals, either without long-term health damages or with severe health problems, might be less motivated to participate. Moreover, a family history of cancer might influence the willingness to participate, and the statistical power might be limited by the sample size of available former childhood cancer cases. However, the invitations to this study included the maximum number of former childhood cancer patients registered in the German Childhood Cancer Registry that met the inclusion criteria. The recruitment of living patients several years after their diagnosis for the study further limited our analysis to particular patients that suffered from first and second malignancies with a good prognosis. The source population of hospital-based, cancer-free controls is regionally limited to the rural and urban areas around the University Medical Center in Mainz, while population-based cases were recruited all over Germany. However, we do not expect any major differences in the source populations since we expect that neither the interplay between hereditary dispositions and radiation nor cancer have any causal effect on hospitalization after an accident in the Mainz area. Thus, restricting the majority to these controls is equivalent to taking a simple random sample of the original population [74]. In addition, it is known that participation decreases in populations with lower education as well as in very high-income groups. Even though there is no information on socioeconomic status for nonparticipants, we were able to compare the available information of the nonparticipants with the obtained information of the participants. The distribution of sex, age, and age at first diagnosis was similar among participants and nonparticipants and is representative for former childhood cancer patients with these diagnoses in Germany [32].

### Conclusions

To our knowledge, this is the first molecular-epidemiological study on radiation, childhood cancer, and second primary cancer providing a large number of primary fibroblasts from skin biopsies of well-characterized and carefully matched participants for irradiation experiments. In this study, we were able to successfully recruit 441 former SPN and FPN patients from the large survivor cohort of the German Childhood Cancer Registry long after their diagnosis and 150 cancer-free control patients from the Department of Orthopedics and Traumatology of the University Medical Center Mainz. In future projects, the combination of experimental and observational data with a unique study sample, including primary normal somatic cells from former childhood cancer patients and cancer-free controls, will forward research on radiation-related risk factors for childhood cancer, SPNs, and its underlying genetics. Using the gained knowledge from irradiation experiments and analyses on different molecular levels (eg, DNA, RNA, epigenetics), we aim to overcome challenges of personalized childhood cancer therapies and gain insight into the detrimental cellular responses and potential mechanisms of low medically applied radiation doses.

## Appendix

Multimedia Appendix 1 Number of matching groups and time spans for matching between patient groups of participants.

## Abbreviations

CMC: combined multivariate and collapsing
eQTLs: expression quantitative trait loci
FDR: false discovery rate
FPN: first primary neoplasm
GTEx: genotype-tissue expression
GWAS: genome-wide association study
Gy: Gray
ICCC-3: International Classification of Childhood Cancer, Third edition
ICGC: International Cancer Genome Consortium
IPA: Ingenuity Pathway Analysis
OR: odds ratio
PeCan: Pediatric Cancer Genomic Data Portal
Pedican: Pediatric cancer gene database
RVTEST: forest tests
SKAT: sequence Kernel association test
SNP: single nucleotide polymorphism
SNV: single-nucleotide variant
SPN: second primary neoplasm
