# Supplementary material for: Identification of Genetic Predispositions Related to Ionizing Radiation in Primary Human Skin Fibroblasts From Survivors of Childhood and Second Primary Cancer as Well as Cancer-Free Controls: Protocol for the Nested Case-Control Study KiKme
Source: JMIR Res Protoc. 2021 Nov 11;10(11):e32395. doi: 10.2196/32395 (PMC8663494; doi:10.2196/32395)
Supplement: Multimedia Appendix 1 [file resprot_v10i11e32395_app1.docx]

Appendix Table 1. Number of matching groups and time spans for matching between patient groups of participants.

| **Comparison** | **Number of matching groups (N=168)** | | **Percent (%)** | **Cumulative %** |
| --- | --- | --- | --- | --- |
| **Number of matching groups** | | | | |
| 1 SPN^a^, at least 1 FPN^b^, and at least 1 control | | 78 | 46% | 46% |
| 1 SPN^a^ and at least 1 FPN^b^ | | 13 | 8% | 54% |
| 1 SPN^a^ and at least 1 control | | 3 | 2% | 56% |
| At least 1 FPN^b^ and at least 1 control | | 21 | 13% | 69% |
| Only 1 SPN | | 7 | 4% | 73% |
| Only and at least 1 FPN | | 44 | 26% | 99% |
| Only and at least 1 control | | 2 | 1% | 100% |
| **Difference in calendar year of first diagnosis of SPNs^a^ and FPNs^b^** | | | | |
| < 1 year | 17 | | 19% | 19% |
| 1 year to < 2 years | 44 | | 48% | 67% |
| 2 years to < 3 years | 20 | | 22% | 89% |
| 3 years to < 4 years | 6 | | 7% | 96% |
| 4 years to < 5 years | 1 | | 1% | 97% |
| 5 years to < 6 years | 2 | | 2% | 99% |
| 7 years | 1 | | 1% | 100% |
| **Difference in age at first diagnosis of SPNs^a^ and FPNs^b^** | | | | |
| < 1 year | 26 | | 29% | 29% |
| 1 year to < 2 years | 42 | | 46% | 75% |
| 2 years to < 3 years | 14 | | 15% | 90% |
| 3 years to < 4 years | 6 | | 7% | 97% |
| 4 years | 3 | | 3% | 100% |
| **Difference in age at recruitment of SPNs^a^ and FPNs^b^** | | | | |
| < 1 year | 27 | | 30% | 30% |
| 1 year to < 2 years | 23 | | 25% | 55% |
| 2 years to < 3 years | 23 | | 25% | 80% |
| 3 years to < 4 years | 12 | | 13% | 93% |
| 4 years to < 5 years | 3 | | 3% | 97% |
| 5 years | 3 | | 3% | 100% |
| **Difference in age at recruitment of SPNs^a^ and controls** | | | | |
| < 1 year | 24 | | 30% | 30% |
| 1 year to < 2 years | 32 | | 40% | 70% |
| 2 years to < 3 years | 15 | | 19% | 89% |
| 3 year to < 4 years | 5 | | 6% | 95% |
| 4 years to < 5 years | 2 | | 2% | 97% |
| 5 years | 1 | | 1% | 98% |
| 9 years and 10 years | 2 | | 2% | 100% |

^a^ SPNs: second primary neoplasms.

^b^ FPNs: first primary neoplasms.
